# Molecular Dynamics Study on Hygrothermal Aging Mechanisms of Silicone Rubber

**DOI:** 10.3390/ma18225072

**Published:** 2025-11-07

**Authors:** Xiangqi Meng, Kaixun Liu, Liyuan Yang, Huicong Liu, Haining Chen, Weiping Li

**Affiliations:** 1 State Key Laboratory of Tropic Ocean Engineering Materials and Materials Evaluation, School of Materials Science and Engineering, Beihang University, Beijing 100191, China; 2 Aviation Key Laboratory of Science and Technology on Advanced Corrosion and Protection for Aviation Material, AECC Beijing Institute of Aeronautical Materials, Beijing 100095, China

**Keywords:** silicone rubber, molecular dynamics simulation, hygrothermal aging, mechanical properties, thermal decomposition

## Abstract

Silicone rubber, primarily composed of polydimethylsiloxane (PDMS) chains, is widely used in sealing materials due to its excellent flexibility and durability. Its performance is significantly affected by environmental conditions, with humid-heat aging being a major factor of degradation. In this study, molecular dynamics simulations were conducted to systematically investigate the effects of water and temperature on PDMS at the molecular scale. The glass transition temperature (T_g_) and free volume distribution were analyzed to evaluate the mobility of polymer chains under hydrated conditions. Mechanical simulations (including tensile and compressive deformation) indicate that the combined effect of elevated temperature and moisture significantly accelerates the degradation of rubber properties. Thermal decomposition simulations indicate that, under high-temperature and humid conditions, PDMS main chains gradually break into small molecules, with free radical reactions further promoting the aging process. The results elucidate the molecular mechanisms underlying silicone rubber performance deterioration under the coupled action of water and temperature, providing a theoretical basis for service-life prediction and durability design of sealing materials.

## 1. Introduction

Rubber sealing materials are widely used in aerospace, automotive, and energy industries due to their high elasticity, excellent sealing performance, and ability to withstand complex service environments [1,2]. The long-term reliability of seals is directly related to the safety and service life of sealing systems. However, during long-term storage or service, rubber inevitably experiences the combined effects of environmental factors such as temperature, oxygen, humidity, and mechanical stress, leading to the degradation of its physical and chemical properties [3]. Among various rubber materials, silicone rubber is extensively used in high-performance sealing applications owing to its excellent heat resistance, chemical stability, and flexibility over a wide temperature range [4,5,6,7]. Nevertheless, silicone rubber remains sensitive to coupled aging factors such as heat, oxygen, and humidity, which can cause chain scission, crosslinking, and network rearrangement, resulting in significant deterioration of its mechanical and sealing properties [8]. Conventional experimental methods, such as thermal aging and hygrothermal accelerated aging tests, have been widely employed to evaluate the degradation of rubber properties. These experiments can provide macroscopic information, including tensile strength, elongation at break, compression set, and changes in chemical composition [9,10]. However, such methods have limitations in revealing the molecular-scale mechanisms of aging, such as free volume distribution, chain dynamics, and water molecule permeation or interfacial effects [11]. Moreover, commonly used lifetime prediction models—such as the Arrhenius model, time–temperature superposition principle (TTSP), and diffusion-limited oxidation (DLO) models—largely rely on empirical fitting and often fail to accurately describe the complex aging behavior under the coupled effects of temperature, oxygen, and humidity [12].

In recent years, molecular dynamics (MD) simulations have emerged as a powerful computational tool, providing atomistic insights into rubber aging mechanisms [13,14,15,16,17]. By tracking the time-dependent trajectories of molecular systems, MD simulations enable quantitative analyses of key parameters such as fractional free volume (*FFV*), mean square displacement (*MSD*), diffusion coefficients, and radius of gyration (*R_g_*) [18,19,20]. These microscopic parameters are critical for understanding how environmental factors, including temperature, humidity, and reactive species, influence polymer network structure and chain segment dynamics. Meanwhile, with the development of advanced reactive force fields, such as ReaxFF, MD simulations are now capable of directly capturing chemical reactions during aging, including chain scission, crosslinking, and hydrolysis [21,22]. Previous studies have demonstrated the potential of MD simulations in rubber aging research. For example, Choi et al. [23] used MD simulations to study polyurethane failure in seawater, employing models with different molecular weights to represent different failure states and uniaxial stretching to compare tensile strengths; Tamir et al. [24] computed the relaxation modulus of fluororubber to provide a molecular-level explanation of viscoelastic behavior; Karuth et al. [25] employed ReaxFF to investigate the degradation of epoxy resins under hygrothermal conditions, revealing the plasticizing effect of water molecules on crosslinked networks and structural rearrangements; Hattemer [26] computed the storage and loss moduli of coarse-grained polymer nanocomposites to analyze viscoelastic changes; Shi et al. [27] investigated the tensile and viscoelastic properties of rubber nanocomposites filled with silica particles using MD, obtaining performance metrics through uniaxial stretching and oscillatory shear simulations. These studies demonstrate that MD simulations not only complement experimental approaches in elucidating microscopic aging mechanisms but also provide critical data for lifetime prediction and performance modeling.

MD simulations, as an important atomistic tool, have been widely employed to investigate the multi-scale properties of silicone rubber systems. Cambiaso et al. [28] developed a transferable coarse-grained polydimethylsiloxane (PDMS) model compatible with the Martini 3 force field, which accurately reproduces the experimentally observed chain conformations and thermodynamic properties, providing a reliable framework for simulating silicone rubber systems under various conditions. Manap et al. [29] applied MD simulations to study the demolding process of PDMS nanostructures in soft lithography, revealing that interfacial interactions and molecular deformation play a critical role in governing the mechanical response of the material. Lou et al. [30] employed reactive force field MD simulations to investigate the structural evolution of PDMS under combined radiation–thermal aging, showing that chain scission, crosslinking, and small-molecule formation at the atomic scale significantly affect the glass transition temperature and chain mobility. Their study provides molecular-level insights into the degradation mechanisms of silicone rubber and contributes to understanding its temperature-dependent mechanical behavior. Collectively, these works demonstrate that MD simulations can effectively capture the microstructural evolution and mechanical response of silicone rubber systems.

Therefore, this study focuses on silicone rubber and systematically investigates its aging behavior using molecular dynamics simulations. Both conventional and reactive force fields are employed to describe intermolecular interactions, and polymer models as well as polymer–water mixed models are constructed under different temperature and moisture conditions. By analyzing the evolution of polymer chain structures and their dynamic behavior, this work aims to reveal the degradation mechanisms of silicone rubber at the atomic scale and provide theoretical guidance for predicting material performance and evaluating service life.

## 2. Computational Methods

### 2.1. Model Construction and Simulation Procedures

**Figure 1:**
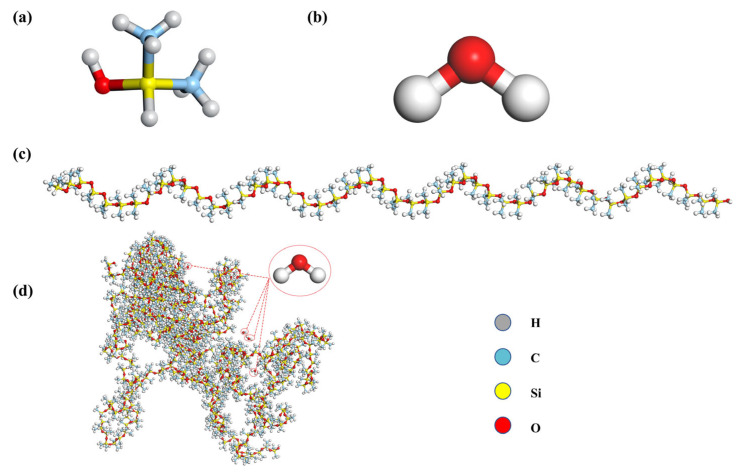
Molecular structure models of PDMS and water systems: (**a**) The monomer unit of PDMS; (**b**) water molecule; (**c**) single PDMS chain; (**d**) PDMS/water mixture (1% water content).

To investigate the interactions between polydimethylsiloxane (PDMS) and water molecules, all-atom molecular dynamics models were constructed. The simulation systems include pure PDMS and PDMS/water mixtures with water contents of 1%, 3%, and 5%, corresponding to 20, 60, and 100 water molecules, respectively. The molecular structures are shown in Figure 1: Figure 1a shows the monomer unit of PDMS; Figure 1b shows the water molecule model; Figure 1c depicts a single PDMS chain, polymerized from siloxane units with a degree of polymerization of 50; Figure 1d illustrates the mixed system consisting of 10 PDMS chains and 20 water molecules, representing the 1% water-containing PDMS system; and the inset provides a close-up view, clearly showing the incorporation of water molecules. Other water content systems were generated using the same approach by adjusting the number of water molecules. Polymer chains were generated using the EMC software (version 9.4.4) [31], with polymer interactions described by the PCFF force field [32], while water molecules were modeled using the SPC/E model. Non-bonded interactions between PDMS and water were described by the Lennard–Jones potential, with parameters taken from the literature [33,34]. Long-range electrostatic interactions were calculated using the PPPM method with a cutoff of 10 Å and an accuracy of 10^−4^. This modeling approach captures the distribution and migration of water molecules in PDMS under humid conditions.

During the modeling process, polymer chains were initially placed randomly in the simulation box. To eliminate atomic overlaps, a soft cosine potential was applied to gradually separate overlapping atoms, followed by NVT equilibration for 250 ps to obtain a reasonable initial structure. Energy minimization was then performed using the conjugate gradient method to optimize residual structural inconsistencies and ensure a stable starting configuration. Pre-equilibration involved a heating–equilibration–cooling–equilibration cycle from 298 K to 500 K, with each stage lasting 250 ps (total 1 ns), allowing polymer chains to relax fully. Subsequently, the system was equilibrated under the NPT ensemble at 298 K for 1 ns. Periodic boundary conditions were applied to reduce boundary effects and computational cost. After equilibration, the system energy stabilized, and the siloxane density was approximately 0.95 g/cm^3^, consistent with reported values.

For simulation parameters, the SHAKE algorithm was used to constrain the bond lengths and angles of water molecules, and Lennard–Jones cross-interaction parameters were calculated using the Lorentz–Berthelot rules. The temperature was controlled using Nosé–Hoover thermostat with coupling constant of 0.1 ps. The pressure was controlled by Nosé–Hoover barostat with coupling constant of 1 ps. The pyrolysis process was modeled using the ReaxFF reactive force field to describe bond breaking and formation events during chemical reactions. All MD simulations were performed using the open-source LAMMPS package (version 29 August 2024, Update 1) [35]. The free volume was calculated with the aid of the Multiwfn program (version 3.8) [36,37].

In this study, the mechanical properties of the polymer were systematically investigated using molecular dynamics simulations. Uniaxial tensile and recovery simulations were performed at 1 atm with a strain rate of 1 × 10^10^ s^−1^. Subsequently, compression–recovery simulations with a compression ratio of approximately 30% were conducted, and the permanent compressive deformation was calculated by comparing the thickness before and after compression. All tensile and compression simulations were conducted at various temperatures to reveal the influence of temperature on the mechanical response of the material. At each temperature, independent tensile and compressive simulations were performed along the x, y, and z directions, and the results were averaged to minimize the influence of anisotropy in the system.

### 2.2. Definition and Calculation of Physical Quantities

To comprehensively characterize the microscopic and macroscopic behaviors of the polymer systems, several physical quantities were calculated from the molecular dynamics trajectories. These include total energy, radius of gyration, velocity autocorrelation function, mean square displacement, fractional free volume, orientation function, mean square end-to-end distance, radial distribution function, and compression set.

1. Total Energy (*E_potential_*)

The total potential energy of the system was obtained directly from the force field and consists of bonded and non-bonded contributions. For the PCFF force field, the total energy consists of bond stretching energy (arising from variations in bond lengths), angle bending energy (arising from changes in bond angles), torsional energy associated with dihedral angles, out-of-plane vibrational energy, and non-bonded interactions, including van der Waals and Coulombic contributions.

(1) $${\;E}_{potential}=E_{bond}+E_{angle}+E_{dihed}+E_{improper}+E_{vdwl}+E_{coul}$$

Monitoring the energy evolution during equilibration and deformation provides insight into structural stability and energy dissipation mechanisms.

2. Radius of Gyration (*R_g_*)

The radius of gyration describes the spatial extent and conformational compactness of polymer chains:

(2) $${\;R}_{g}=\sqrt{\frac{1}{N}\sum_{i=1}^{N}{\left(r_{i}-r_{cm}\right)}^{2}}\;$$

where *r_i_* is the position of atom *i* and *r_cm_* is the center of mass of the chain. Changes in *R_g_* reflect polymer chain relaxation and aggregation behavior.

3. Velocity Autocorrelation Function (VACF)

The VACF quantifies the correlation between atomic velocities at different times and reflects the dynamic mobility of atoms:

(3) $${\;C}_{v}\left(t\right)=\frac{\left\langle v\left(0\right)v\left(t\right)\right\rangle }{\left\langle v{\left(0\right)}^{2}\right\rangle }\;$$

where *v*(0) is the velocity vector of an atom at the reference time *t* = 0 and *v*(*t*) is the velocity vector of an atom at time.

The decay behavior of *C_v_*(*t*) indicates the transition from ballistic to diffusive motion.

4. Mean Square Displacement (*MSD*)

The *MSD* describes the average displacement of atoms over time and is a key measure of diffusion:

(4) $$MSD\left(t\right)=\left\langle |r\left(t\right)-r\left(0\right){|}^{2}\right\rangle \;$$

where *r_i_*(*t*) is the position vector of atom *i* at time t and *r_i_*(0) is its position at the reference time *t* = 0.

5. Fractional Free Volume (*FFV*)

The *FFV* represents the fraction of unoccupied space within the polymer matrix and was calculated using the Multiwfn program:

(5) $$FFV=\frac{V_{total}-V_{occupied}}{V_{total}}\;$$

A larger *FFV* indicates more free space available for molecular motion and diffusion.

6. Orientation Function

The orientation function quantifies the alignment degree of polymer chains along a specific direction, defined as:

(6) $$f=\frac{3\left\langle {cos}^{2}\right.\left.\theta \right\rangle -1}{2}\;$$

where *θ* is the angle between the chain axis and the reference direction. *f* = 0 indicates random orientation, while *f* = 1 represents perfect alignment.

7. Mean Square End-to-End Distance (*R*^2^*_ee_*)

The mean square end-to-end distance characterizes chain conformation and flexibility:

(7) $${\;R^{2}}_{ee}=\left\langle \left|r_{head}\right.-{\left.r_{tail}\right|}^{2}\right\rangle \;$$

Variation in *R*^2^*_ee_* reflects chain stretching and entanglement during deformation.

8. Radial Distribution Function (*g*(*r*))

The radial distribution function (RDF) is a statistical quantity that characterizes the relative position and distance distribution between atoms or molecules in a multi-atomic system. It describes the probability density of finding other atoms or molecules at a distance *r* from a reference atom or molecule. The radial distribution function *g*(*r*) can be expressed as follows:

(8) $$\;g\left(r\right)=\frac{N}{V}\times \frac{1}{\rho }\times \frac{dN}{dr}\;$$

Here, *N* denotes the number of atoms or molecules within a distance *r*, *V* represents the system volume, ρ is the average number density of atoms or molecules, and *dN*/*dr* indicates the rate of change in the number of atoms or molecules at a distance r.

9. Compression Set (*CS*)

The compression set quantifies the permanent deformation after compressive loading and recovery:

(9) $$CS=\frac{h_{0}-h_{r}}{h_{0}-h_{c}}\times 100\%\;$$

where *h*_0_ is the initial thickness, *h_c_* is the compressed thickness, and *h_r_* is the recovered thickness. A smaller *CS* value indicates better elastic recovery.

## 3. Results and Discussion

### 3.1. Validation of System Equilibration

**Figure 2:**
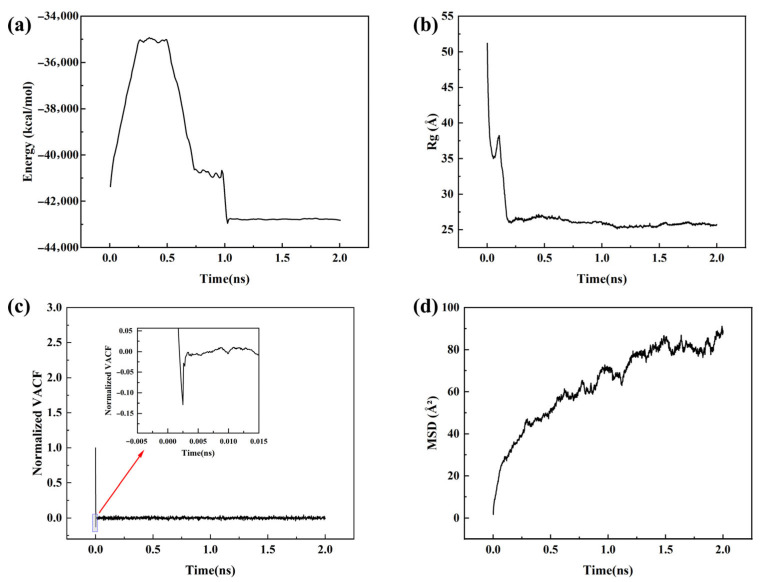
Temporal evolution of key parameters during system equilibration: (**a**) total energy; (**b**) radius of gyration; (**c**) velocity autocorrelation function; (**d**) mean square displacement.

Energy is a key indicator of system equilibration, as it converges rapidly and directly reflects the transition from a high-energy configuration to a thermodynamically stable state. The radius of gyration (*R_g_*), defined as the root-mean-square distance of atoms from the center of mass, characterizes the conformational compactness of polymer chains. A stable *R_g_* indicates that the chain conformation remains unchanged, providing strong evidence of structural equilibration. The velocity autocorrelation function (VACF) describes the temporal correlation of particle velocities; its rapid decay and stabilization near zero suggest that the dynamic behavior of the system is no longer correlated with the initial configuration, demonstrating dynamic equilibration. The mean square displacement (*MSD*) reflects particle diffusivity and exhibits a stable linear increase under equilibrium, with its slope directly related to the diffusion coefficient, enabling reliable extraction of dynamic properties. Figure 2 presents the parameter variations during the 2 ns equilibration stage. The results indicate that energy, *R_g_*, and VACF have stabilized, while the slope of MSD remains steady, confirming that the system has reached equilibrium and is ready for subsequent statistical property calculations.

### 3.2. Evolution of Characteristic Temperature and Structural Response

**Figure 3:**
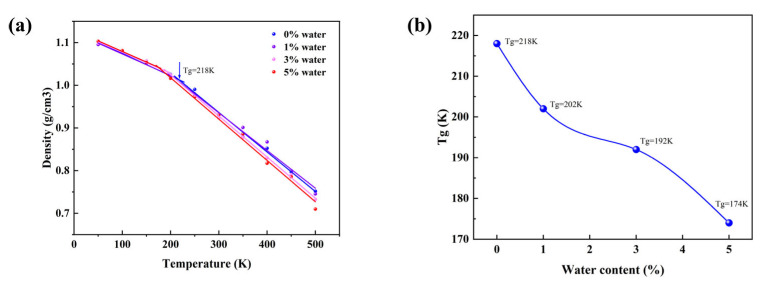
(**a**) Variation in density with temperature for PDMS systems at different water contents. (**b**) The glass transition temperature (T_g_) of PDMS systems with different water contents.

The glass transition temperature (T_g_) reflects the dynamical transition of polymer chain segments from frozen to relaxed states and serves as an important parameter to validate the reliability of molecular simulation models. In this study, the T_g_ was determined from the inflection point of the density–temperature curve. Figure 3 presents the variation in density with temperature and the corresponding T_g_ for PDMS systems with different water contents. The results show that the T_g_ of the dry PDMS model is approximately 218 K. With the introduction of 1%, 3%, and 5% water molecules, the T_g_ decreases to about 202 K, 192 K, and 174 K, respectively, exhibiting a progressive reduction with increasing water content. This trend indicates that water molecules significantly weaken the intermolecular interactions between polymer chains and increase the free volume of the system, thereby facilitating chain relaxation and mobility at lower temperatures. It should be noted that the cooling rate in molecular simulations is typically much higher than that in experiments, which may result in an overestimation of the absolute T_g_. Nevertheless, under identical cooling conditions, the relative comparison reasonably captures the effect of water on the glass transition behavior. Overall, the presence of water molecules promotes chain relaxation at the molecular level, leading to a gradual decrease in T_g_ with increasing water content.

**Figure 4:**
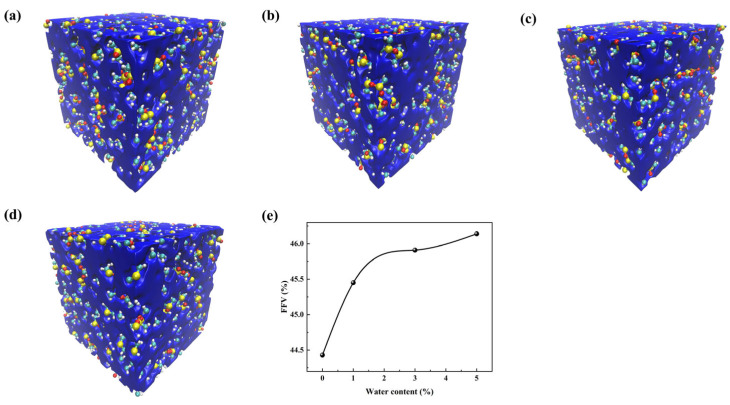
Free volume analysis of PDMS systems at 298K with different water contents: (**a**–**d**) *FFV* visualizations for 0%, 1%, 3%, and 5% water; (**e**) variation in *FFV* with water content.

Further calculations of the free volume fraction at 298 K were performed for systems with different water contents (Figure 4). To visually illustrate the distribution of free volume, FFV visualizations were generated for each system (Figure 4a–d), where blue regions represent free volume. It can be observed that, with the introduction of water, the number of free volume regions increases and their distribution becomes more uniform. Quantitative results (Figure 4e) show that as the water content increases from 0% to 5%, the free volume fraction rises from 44.4% to 46.1%. This combined quantitative and visual analysis indicates that water molecules increase the free volume, providing more space for polymer segment motion and offering a microscopic explanation for the observed decrease in T_g_.

### 3.3. Molecular Dynamics Simulation of Tensile Behavior

**Figure 5:**
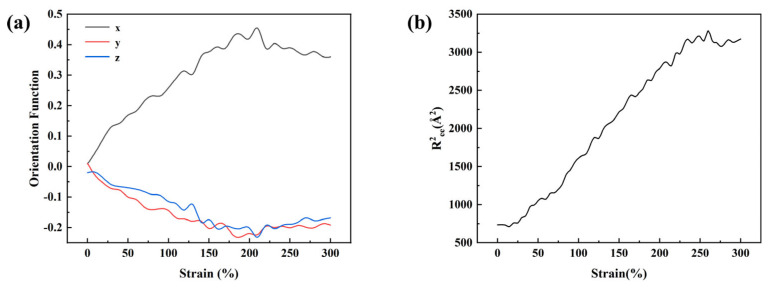
(**a**) Evolution of molecular chain orientation with strain. (**b**) Variation in mean square end-to-end distance with strain.

To investigate the mechanical properties of the system, uniaxial tensile simulations were performed. Under a prescribed strain rate, deformation was applied by stretching the simulation box along a single direction. To improve the reliability of the results, tensile loading was conducted along the x, y, and z directions, and the averaged values were reported. The tensile process was carried out in the NPT ensemble, where the two directions perpendicular to the loading axis were maintained at constant pressure. The orientation function and mean-squared end-to-end distance (*R^2^_ee_*) were employed to characterize the chain orientation and conformational evolution during deformation, which are crucial for understanding the microscopic deformation mechanisms of polymers. Figure 5 presents the changes in chain orientation (a) and *R^2^_ee_* (b) with strain for the water-free system under x-direction loading at room temperature. The results indicate that, with increasing strain, the chains gradually orient along the tensile direction: the orientation function in the x-direction rises significantly, suggesting preferential chain alignment along the loading axis, while those in the y and z directions decrease, reflecting the gradual loss of random orientation in the transverse directions. This demonstrates that polymer chains evolve from a disordered to an ordered state under external loading. Meanwhile, the *R^2^_ee_* continuously increases with strain, indicating that the chains extend from a coiled state to a more stretched conformation. Overall, these findings reveal the chain orientation and extension process of polymers under tensile loading, providing molecular-level insights into their macroscopic mechanical response.

**Figure 6:**
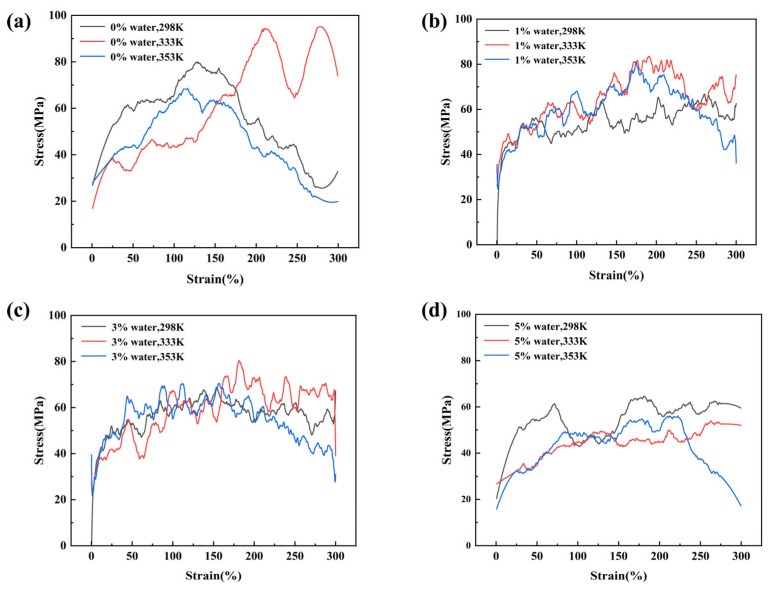
Tensile stress–strain curves of the same system at different temperatures: (**a**) 0% water; (**b**) 1% water; (**c**) 3% water; (**d**) 5% water.

Figure 6 presents the stress–strain curves of PDMS systems under different temperatures and water contents, where Figure 6a–d correspond to water contents of 0%, 1%, 3%, and 5%, respectively. It should be noted that the applied strain rate in molecular dynamics simulations is much higher than in experiments, and thus the stress–strain curves cannot be directly compared with experimental results on a quantitative basis; nevertheless, they provide valuable qualitative insights. The results show that all systems undergo three characteristic stages during tensile deformation: linear elasticity, strain hardening, and failure, which are consistent with the typical mechanical responses of PDMS-based elastomers observed in molecular dynamics simulations. Owing to the high strain rate, the systems exhibit elevated stress levels and elastic moduli. With increasing temperature, the stress drop in the failure stage becomes more pronounced, indicating that higher temperatures enhance chain mobility and facilitate chain slippage. Moreover, at a fixed temperature, increasing water content also accelerates the stress drop during failure, suggesting that water molecules act as plasticizers and weaken the intermolecular interactions between PDMS chains.

**Figure 7:**
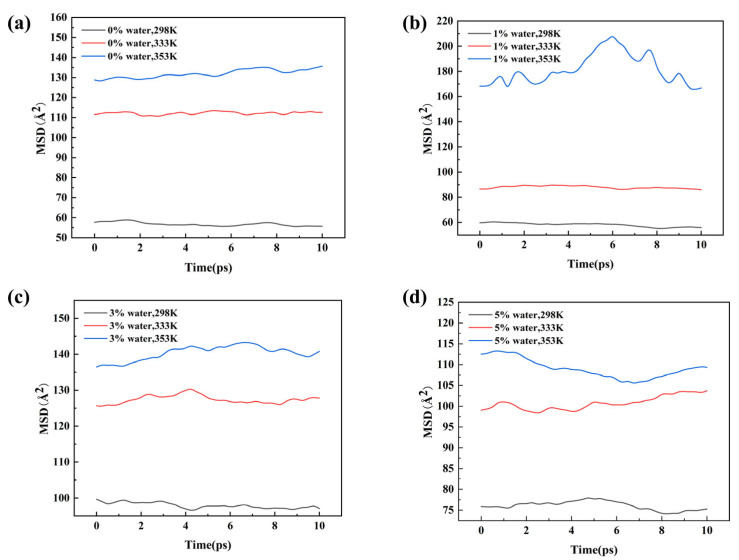
Mean square displacement (MSD) curves of the same system at different temperatures: (**a**) 0% water; (**b**) 1% water; (**c**) 3% water; (**d**) 5% water.

Figure 7 presents the mean square displacement (*MSD*) as a function of time, used to evaluate the effect of temperature on molecular mobility. The analysis is based on the last 10 ps of the simulation trajectories. The *MSD* curves indicate that temperature is the primary factor controlling molecular motion in the system. In all four systems with different water contents, *MSD* consistently increases with temperature, reflecting faster diffusion of molecular segments at higher temperatures and demonstrating that increased thermal energy directly enhances segmental mobility. The overall behavior follows the Arrhenius relation, indicating that elevated temperatures provide sufficient activation energy to overcome motion barriers, thereby significantly promoting Brownian motion and molecular diffusion.

**Figure 8:**
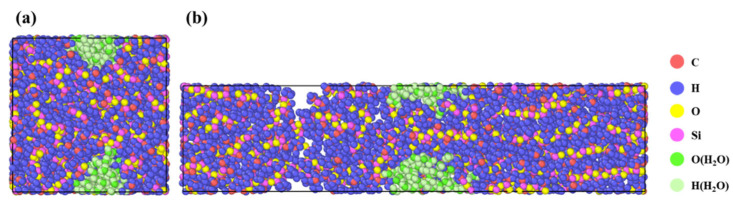
Molecular models under different tensile states: (**a**) initial state (strain 0%); (**b**) stretched state (strain 200%).

After constructing molecular models with different compositions, in addition to calculating relevant physical quantities and simulating mechanical properties, the molecular trajectories were also analyzed. Figure 8 presents the simulation snapshots of the system at the initial strain (0%) and at 200% strain, where water molecules are shown in green (hydrogen and oxygen atoms). This figure is intended to provide an intuitive analysis of the influence of water molecules on the polymer matrix. The results indicate that water molecules tend to spontaneously aggregate into clusters when incorporated into the polymer, and during tensile deformation, these clusters evolve into defect-rich regions, thereby reducing the overall mechanical performance of the system.

**Figure 9:**
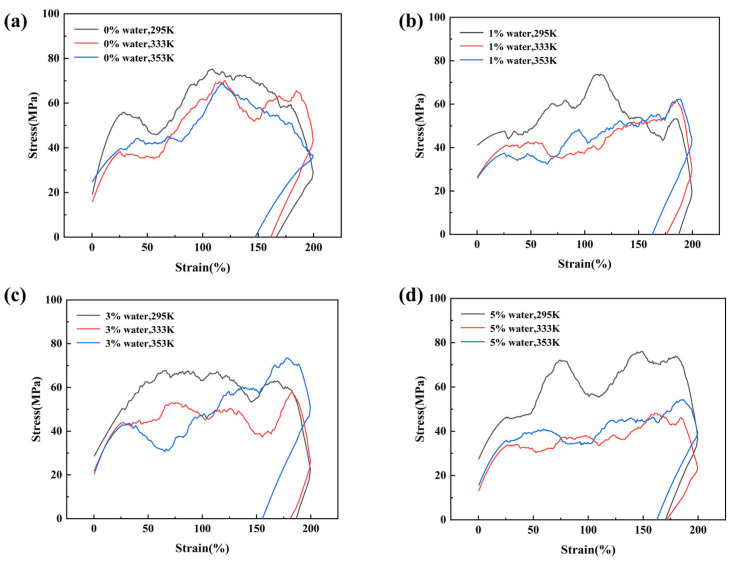
Stress–strain curves of the same system during tensile–recovery at different temperatures: (**a**) 0% water; (**b**) 1% water; (**c**) 3% water; (**d**) 5% water.

In the preceding tensile simulations, we systematically examined the effects of temperature and water content on the mechanical response of the system. Building upon these results, tensile–recovery simulations were further performed to evaluate the elastic recovery capacity and permanent deformation behavior. Figure 9 presents the stress–strain curves of the same systems under different temperatures during the tensile–recovery process. In the simulations, the systems were first stretched to twice their initial length and then unloaded at the same strain rate. The recovery curves intersect with the strain axis, indicating the occurrence of permanent deformation. A detailed analysis reveals that the tensile strength decreases with increasing temperature across all systems, but the magnitude of this reduction varies with water content: the anhydrous system exhibits the highest temperature sensitivity, whereas in systems with 3% and 5% water, the tensile strength at 333 K and 353 K remains nearly unchanged, though still significantly lower than that at room temperature. Moreover, permanent deformation exhibits a clear increasing trend with temperature across all systems. These results suggest that the combined effect of elevated temperature and water content may accelerate the irreversible structural evolution of the polymer system.

**Figure 10:**
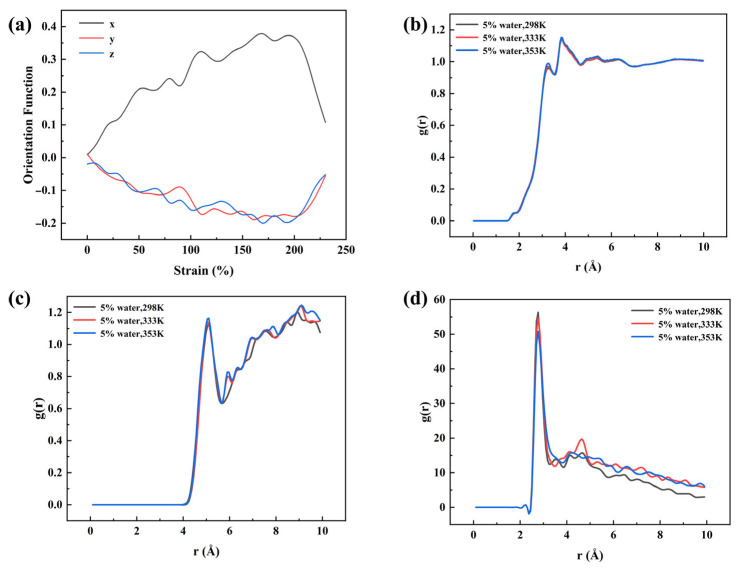
(**a**) Variation in chain orientation during tensile–recovery. (**b**) Radial distribution function of all atoms in the 5% water-containing system at different temperatures. (**c**) Radial distribution function of Si–Si pairs in PDMS chains at different temperatures. (**d**) Radial distribution function of O–O pairs in water at different temperatures.

Figure 10a illustrates the evolution of chain orientation during the tensile–recovery process. During stretching, the orientation function along the loading direction increases progressively, indicating that polymer chains gradually align along the tensile axis. During recovery, the chain orientation decreases as strain is released but does not return to the initial level, reflecting irreversible chain rearrangements in the system. Figure 10b presents the radial distribution function (RDF) of all atoms in the system with 5% water content, characterizing the spatial distribution of atoms as a function of interatomic distance. At 298 K and 333 K, the RDF curves overlap closely, suggesting stable local structures within this temperature range. In contrast, at 353 K, the first RDF peak becomes significantly higher, indicating enhanced local atomic aggregation and a more compact structure. Combined with the observed increase in permanent deformation, this suggests that when water content reaches a certain level and the temperature rises to a critical range, the intensified motion of water molecules promotes local clustering and irreversible chain rearrangements, thereby amplifying permanent deformation. Figure 10c shows the RDF of Si–Si pairs in PDMS chains, where the first peak increases with temperature, indicating local chain contraction and the formation of denser short-range order at elevated temperatures. Figure 10d presents the O–O RDF of water molecules, with the first peak decreasing as temperature rises, suggesting reduced local clustering and enhanced mobility of water molecules. This trend complements the behavior observed in the Si–Si RDF, supporting the conclusion that water mobility at high temperatures facilitates irreversible chain rearrangement and amplifies permanent deformation.

### 3.4. Molecular Dynamics Simulation of Compression Behavior

**Figure 11:**
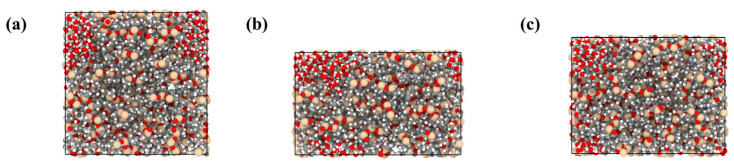
Molecular models showing thickness changes during compression–recovery: (**a**) original thickness; (**b**) compressed thickness; (**c**) recovered thickness.

**Figure 12:**
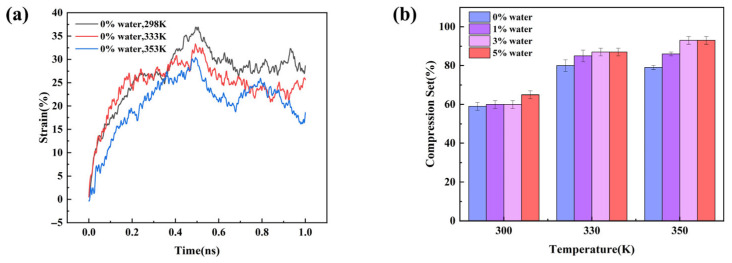
(**a**) Time evolution of compression–recovery strain in the water-free system at different temperatures. (**b**) Compression set under different conditions.

In this study, compression–recovery simulations were performed on the molecular models. Taking the anhydrous PDMS system as an example, Figure 11 illustrates the evolution of the simulation box dimensions during the process. During compression, the box thickness along the loading direction decreased significantly, forcing polymer chains to approach each other and exhibit a certain degree of orientation and stacking. In the subsequent recovery stage, the box thickness gradually increased but did not fully return to its initial value, indicating irreversible permanent deformation. At the molecular level, some chain segments underwent conformational rearrangements during compression and failed to fully recover during recovery. Figure 12a further shows the time-dependent evolution of compression–recovery strain at different temperatures. It can be seen that when the compression ratio exceeds 30%, the system fails to return to its original dimensions even though the compression and recovery stages have the same duration. This observation is consistent with the irreversible molecular conformational changes seen in Figure 11, indicating a close correlation between macroscopic irreversible deformation and molecular-level conformational rearrangements. Figure 12b presents the compression set of the material under different temperature and moisture conditions. It is evident that the compression set increases markedly with rising temperature, indicating that the elastic recovery of the material is significantly reduced at elevated temperatures. This can be attributed to the enhanced chain mobility at higher temperatures, which facilitates irreversible slippage and rearrangement of polymer chains, leading to larger permanent deformation. In addition, the compression set also increases with higher moisture content. At the same temperature, the 0% water system exhibits the lowest compression set, whereas the 5% water system shows the highest. For instance, at 350 K, the compression set rises from approximately 79% in the dry system to nearly 90% in the high-moisture system. This suggests that moisture acts as a plasticizer, weakening intermolecular interactions and further promoting irreversible deformation. Overall, temperature and moisture exhibit a synergistic effect, substantially reducing the compressive recovery ability of the material.

### 3.5. Molecular Dynamics Analysis of Thermal Decomposition

**Figure 13:**
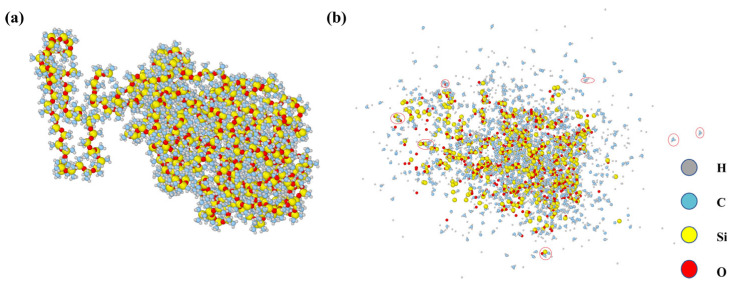
Molecular models: (**a**) rubber system before pyrolysis; (**b**) rubber system after pyrolysis.

Rubber materials undergo pyrolysis at elevated temperatures, which is a critical issue in understanding their service stability and failure mechanisms. Molecular dynamics simulations of the pyrolysis process allow the fracture behavior of polymer chains and the formation of decomposition products to be directly visualized at the molecular scale, thereby providing theoretical insights into pyrolysis kinetics and guiding experimental analysis. Figure 13 illustrates the structural evolution of the rubber system before and after pyrolysis. To directly reflect the spatial distribution of molecules, the real configurations without periodic boundary reconstruction are presented. As shown in Figure 13a, prior to pyrolysis, the system mainly consists of aggregated long chains with relatively intact connectivity. In contrast, Figure 13b reveals that after pyrolysis, the long chains are fragmented, and the system is broken into more dispersed small molecules or short chains, indicating that chemical bonds are cleaved and macromolecules gradually degrade into small-molecule products. The red circles in the figure highlight the methyl groups, methane molecules, and short-chain fragments. This transformation clearly reflects the essential process of polymer-to-small-molecule conversion during pyrolysis.

**Figure 14:**
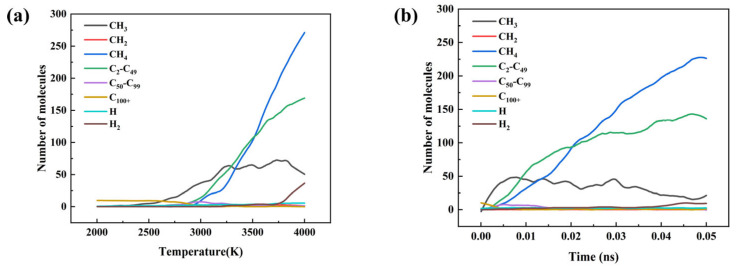
Variation in the number of pyrolysis product molecules in the water-free rubber system: (**a**) molecular count versus temperature; (**b**) molecular count versus time at 3000 K.

Figure 14 illustrates the evolution of decomposition products in the rubber system as a function of temperature and time. During the heating process (Figure 14a), significant pyrolysis occurs at approximately 2800 K, characterized by chain scission and the formation of small molecules. Methane (CH_4_) is generated in large quantities, while fragments containing 2–49 carbon atoms increase markedly. In addition, methyl radicals (CH_3_•) first accumulate rapidly and then decrease, indicating that they act as key intermediates but are subsequently consumed or transformed through secondary reactions. The isothermal simulation at 3000 K (Figure 14b) further highlights the kinetic features of the process, where methane molecules increase at an approximately constant rate, enabling the estimation of reaction rate constants from the linear growth region. Overall, Figure 14 demonstrates that rubber pyrolysis proceeds through a sequential process of chain scission, radical generation, and the accumulation of stable small molecules.

These microscopic pyrolysis characteristics are closely correlated with the macroscopic deterioration of rubber during aging. Chain scission reduces the molecular weight and disrupts the polymer network, leading to significant losses in mechanical strength and elasticity. The continuous formation and volatilization of small molecules generate microstructural defects, accelerating the decline of mechanical and sealing performance. Moreover, secondary radical reactions may trigger localized crosslinking, resulting in hardening and embrittlement. Thus, the molecular-level mechanisms revealed by the pyrolysis simulations not only capture the product evolution but also provide a fundamental explanation for the performance degradation of rubber under long-term service or high-temperature conditions.

**Figure 15:**
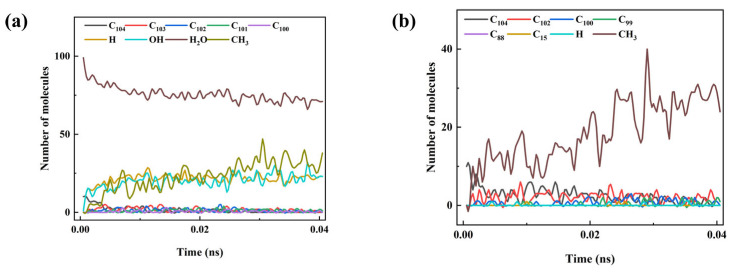
Time evolution of the number of pyrolysis product molecules at 3000 K for different systems: (**a**) rubber–water mixture; (**b**) pure rubber.

Figure 15 presents the temporal evolution of pyrolysis products in the polymer system and the polymer–water mixture at 3000 K, where Figure 15a corresponds to the polymer–water mixture and Figure 15b represents the pure polymer system. It can be observed that in the mixed system, the cleavage of Si–C bonds is more pronounced, leading to the formation of a large number of CH_3_ radicals, which are subsequently converted into methane or methanol through secondary reactions. As the reaction proceeds, the number of water molecules in the mixture gradually decreases, indicating that they are not inert but actively participate in radical-mediated reactions, thereby influencing the final product distribution. In contrast, the pure polymer system exhibits relatively slower chain scission, resulting in fewer small-molecule products. Overall, the introduction of water molecules not only accelerates the cleavage of long polymer chains but also significantly alters the product composition and kinetic characteristics of the pyrolysis process.

## 4. Conclusions

This study, based on molecular dynamics simulations, systematically elucidates the effects of water molecules and temperature on the structural and mechanical properties of PDMS systems. Equilibrium verification using energy, radius of gyration, VACF, and *MSD* confirmed the reliability of the models and provided a solid foundation for subsequent analyses. The results indicate that the incorporation of water molecules significantly reduces the glass transition temperature of PDMS by weakening intermolecular interactions and increasing free volume, thereby promoting chain relaxation. In terms of mechanical response, uniaxial tension and tension–recovery simulations revealed that all systems exhibited typical elastomeric characteristics under different water contents and temperatures. However, higher temperatures and increased water content progressively weakened intermolecular forces, leading to reduced strength and enhanced permanent deformation. Compression–recovery simulations further demonstrated the synergistic plasticization effect of water and heat, resulting in a pronounced increase in compression set and limited elastic recovery. Thermal decomposition simulations showed that PDMS chains undergo scission at elevated temperatures, forming small-molecule products, with water molecules not only accelerating chain cleavage but also altering the reaction kinetics and product distribution. Importantly, these findings have practical implications for PDMS-based materials in engineering applications. For instance, understanding the combined effects of temperature and moisture on chain mobility, mechanical strength, and elastic recovery can guide the design and optimization of PDMS for sealing components, coatings, biomedical devices, and electronic materials, where environmental exposure and water absorption critically affect performance and service life. Furthermore, this work highlights several directions for future research. Extending simulations to longer timescales, exploring more complex environmental conditions, or investigating the behavior of PDMS composites and filled systems could provide deeper insights into aging, failure mechanisms, and strategies to enhance durability under operational conditions.

## Data Availability

The original contributions presented in this study are included in the article. Further inquiries can be directed to the corresponding author.
